# The moderation effect of secure attachment on the relationship between positive events and wellbeing

**DOI:** 10.1002/pchj.546

**Published:** 2022-05-18

**Authors:** Ruth Spence, Lisa Kagan, Stephen Nunn, Deborah Bailey‐Rodriguez, Helen L. Fisher, Georgina M. Hosang, Antonia Bifulco

**Affiliations:** ^1^ Department of Psychology Middlesex University London UK; ^2^ King's College London, Social Genetic & Developmental Psychiatry Centre Institute of Psychiatry, Psychology & Neuroscience London UK; ^3^ Centre for Psychiatry, Wolfson Institute of Preventive Medicine, Queen Mary University of London London UK

**Keywords:** attachment, positive life events, psychosocial models, wellbeing

## Abstract

Positive events can reduce depression as well as enhance wellbeing. The role of secure attachment style in moderating the relationship between positive events and wellbeing is examined to further understand wellbeing models. Participants (*n* = 490) included two midlife groups and a student group from the UK. They completed the online Computerized Life Event Assessment Record (CLEAR), a measure of life events, the Vulnerable Attachment Style Questionnaire (VASQ), and the Warwick–Edinburgh Mental Wellbeing Scale (WEMWBS). Age was associated with higher rates of wellbeing and secure attachment style. A significant relationship was found between number of positive events and wellbeing, number of people close, and secure attachment score. Hierarchical multiple regression indicated a significant interaction between secure attachment style, number of positive life events, and wellbeing. Simple slopes analysis demonstrated the association between positive life events and wellbeing was significant for secure attachment (*B* = 1.27, *p* = .003) but not insecure attachment (B = 0.04, non‐significant). This suggests securely attached individuals are better able to take advantage of positive life events than insecurely attached individuals and experience a greater increase in wellbeing.

## INTRODUCTION

Life events are discrete experiences that cause substantial life changes and readjustment for the individual(s) involved. To date, research has focused largely on negative occurrences, these termed severe events, such as divorce and bereavement. This has led to an extensive literature showing how stressful life changes encompassed by severe events are associated with poorer mental health (e.g., Beards et al., 2013; Hosang et al., 2012). Positive life events (e.g., exam achievement, financial gain, or the improvement in an important relationship) are often overlooked. However, they can have positive impacts on mental health (Headey, 2006), increase feelings of subjective wellbeing and life satisfaction (McCullough et al., 2000; Wootton et al., 2017), and are associated with recovery from depression and anxiety (Brown et al., 1992; Leenstra et al., 1995). Psychosocial models of clinical disorder explain the link between life events and psychological disorder through a vulnerability‐provoking agent model; for example, an existing vulnerability (e.g., social isolation, low self‐esteem) interacts with negative life events to produce disorder in a relatively short time frame. This fits with clinicians “5P formulation” including predisposing and precipitating factors (Dallos & Johnstone, 2014). Currently, there is no developed converse model linking psychological robustness, positive life events, and heightened wellbeing. This is hampered by wellbeing outcomes as less time specific for onset/recovery than clinical disorder and because wellbeing outcomes often subsume elements of positive predisposing factors such as optimism or high self‐esteem with a danger of circularity. At best, it could be argued for an “accumulative positivity effect” linked to a heightening of prior psychological robustness. Yet, positive events do have the potential for accurate dating and for objective assessment of their level of likely positivity and other characteristics such as anchoring or fresh start (Bifulco et al., 2021; Brown et al., 1992). Also, attachment style as a known robustness and resilience factor (Kafetsios & Sideridis, 2006) has some differentiation from more general optimism and other wellbeing cognitive factors. Therefore, utilizing secure attachment as a predisposing factor, given its long reach back to childhood (Bowlby, 1988), positive events as precipitating factors, and step increases in wellbeing could form a parallel model of positive mental health.

Insecure attachment is frequently used as a marker of existing vulnerability in the vulnerability‐provoking agent model of disorder (e.g. Abdul Kadir & Bifulco, 2013; Mikulincer & Shaver, 2017). Here, we use secure attachment as a marker of prior psychological robustness in an “accumulative positivity effect” model of wellbeing. Attachment style refers to the behavioral expression of internal working models that develop early in life to determine how individuals will form and maintain relationships with others in adulthood (Bowlby, 1988). Insecure attachment styles are characterized by maladaptive behaviors clustered around anxious styles (such as being clingy and dependent) and avoidant style (overly self‐reliant and avoidant of intimacy; Bifulco, 2014). Psychosocial models show that individuals with insecure styles have lower levels of social support and are less able to use close others to help regulate their emotions (Bifulco & Thomas, 2012; Mikulincer et al., 2003). This applies both to those with anxious and avoidant styles, although there is different interpersonal expression. Those with insecure styles use less adaptive strategies such as suppression or rumination (Fraley & Shaver, 1997; Mikulincer & Orbach, 1995; Saffrey & Ehrenberg, 2007). Thus, insecurely attached individuals are less effective at coping with environmental stressors such as negative life events. However, secure individuals have more sophisticated coping with stressors, as well as better mental health, support, and emotional regulation (Belsky, 2002; Bifulco & Thomas, 2012; Mikulincer et al., 2002). When faced with negative life events, those with secure attachment styles often rely on support‐seeking strategies to aid coping and to reduce negative emotion. They are able to flexibly relate to others, give and seek help, and build close confiding attachments conferring social support (Bowlby, 1988). Therefore, secure attachment style is the most functional and normative style, with links to positive childhood care making it appropriate as a predisposing factor for adult mental health.

However, the effects of secure attachment style on positive functioning have been somewhat neglected in comparison to the psychopathological effects of insecure attachment style. For instance, although there is evidence demonstrating the way positive events are processed may differ as a product of attachment, it tends to be focused on the effects of insecure attachment style. The research suggests that insecurely attached individuals cannot take advantage of positive life events in the same way that securely attached individuals might. For example, insecurely attached individuals are less able to access memories of positive events (Mikulincer, 1998) and underestimate how they good they felt after positive experiences (Gentzler & Kerns, 2006). There are also processing differences within insecure attachment types implied by the different response of anxious and avoidant insecure types to events; for example, attachment avoidance seems to inhibit the processing of positive events less than attachment anxiety (Gentzler et al., 2010; Shaver & Mikulincer, 2008). However, to our knowledge there are no studies demonstrating whether secure attachment interacts with positive life events in regards to increasing wellbeing.

The more recent links of epistemic trust to attachment processes and mentalizing, indicate that where the source of the information is mistrusted, there are barriers to social communication and taking in information (Fonagy et al., 2017a). This has implications for both accessing social support and therapeutic engagement (Fonagy et al., 2017b), and, we would argue, for appraisal of positive as well as negative events. The seminal work by Lazarus and Folkman (1984) on the importance of the individual appraisal of life events regardless of their objective characteristics (Brown & Harris, 1978) invokes the Beckian tendency in those who are vulnerable to attribute negative cognitive and emotional weight to events around pessimism, mistrust, and self‐doubt (Beck, 1967). Whether such vulnerability also creates an inability to perceive the positive in events needs further elucidation.

The goal of this study is to expand existing psychosocial models of wellbeing by exploring the relationship of secure attachment style, positive life events, and wellbeing, and test for moderating effects. Consistent with past research, we predict that there will be a significant relationship between positive life events and wellbeing, such that increases in positive life events will be associated with higher levels of wellbeing. We also predict a significant interaction between positive life events and secure attachment style leading to even greater wellbeing, even when controlling for current level of social support.

## METHODS

### Participants

Participants were individuals who completed a new online measure of life events, the Computerized Life Events Assessment Record (CLEAR) as part of its initial testing and validation (see Bifulco et al., 2019a). It was completed by 490 participants in the UK, most of whom were female (77.1%) and Caucasian (75.6%). This included 75 midlife individuals with prior recurrent clinical depression and 128 matched controls; both groups were followed‐up from the Depression Case Control study (DECC; Korszun et al., 2004) and 287 undergraduate students, which increased the age range and ethnic composition of the sample. Thus, ages were somewhat polarized with the mean age of the midlife groups at 57.4 (*SD* = 8.34, range: 18–75), and of the students 20.23 (*SD* = 3.81, range: 17–46). The Black, Asian, and Minority Ethnic (BAME) participants were mainly in the student group (67%, 191/287). The majority of the midlife groups were middle class with 85% having home ownership, 62% in work, and 45% being either managerial or self‐employed. A sample size of 107 was needed at a 95% confidence interval (95% CI; α = 0.05), assuming a power of 0.8 and a medium effect size (f^2^ = .15).

### Measures

#### Computerized Life Events Assessment Record

CLEAR is based on the Life Events and Difficulties Schedule (LEDS) interview (Brown & Harris, 1978) completed online via a secure website. It collects quantitative and qualitative data regarding demographics (e.g., date of birth, employment), information about close others (e.g., relationship type, supportiveness), and life events. It has been shown to have good psychometric properties, showing high test–retest reliability and good validity (Bifulco et al., 2019a). The test–retest reliability for positive events measured 3–4 weeks apart was moderate (intraclass correlation coefficient [ICC] = .73, 95% CI [0.61–0.82]) (CLEAR; Bifulco et al., 2019a).

Life events are classed using three overarching categories: “Lifestyle” (education, work, housing, money, crime, and geopolitical events), “Health” (illness, pregnancy, and bereavement); and “Relationships” (partner, children, and close others). Participants rate any events within these subsets that may have occurred during the last 12 months, using guidance provided, pull down and multiple choice menus, and benchmarked examples consistent with LEDS scoring. Analysis of the negative life events and depression in this sample are described elsewhere (Bifulco et al., 2019b).

For all events, an overall “positivity” rating was made from 0 =*not at all: no positive implications experienced or expected* to 4 = *very: many positive qualities*, *lasting*, *beyond expectation*, *overcoming obstacles*. A rating of 3 *moderate* or 4 = *very* on positivity were combined into a dichotomized “highly positive event” rating. For this analysis, each individual was given a binary rating (*yes/no)* for having experienced a highly positive event. Additionally, the total number of positive events experienced by each individual was recorded.

CLEAR also records information about close others, defined as those the person can go to for help and support. Respondents were requested to complete this section for up to three people, although they could complete it for more. They were also given the option to record that they are not close to anyone. In addition, the data includes how confiding each relationship is from 1 = *highly* to 4 = *not at all*. The number of people close to each respondent was calculated; additionally each individual's mean confiding score across their close relationships was calculated. For this analysis. Individuals received a binary rating of two or more people close (*yes/no*) as well as the total number of people close.

#### Vulnerable Attachment Style Questionnaire

The Vulnerable Attachment Style Questionnaire (VASQ) is a 22‐item measure that provides a total score of attachment insecurity. Items are scored on a 5‐point Likert scale from 1 = *strongly disagree* to 5 = *strongly agree*. A score of over 57 indicates an insecure attachment style. Its reliability and validity has been established against an investigator‐based interview (Bifulco et al., 2003) and the Cronbach's alpha for the current sample was .83. The VASQ allows for dichotomous scorings of anxious style, avoidant style or secure style. For this analysis, individuals received a binary rating of secure style (*yes/no*) as well as their total attachment score, with lower scoring denoting greater secure attachment (VASQ; Bifulco et al., 2003).

#### Warwick–Edinburgh Mental Wellbeing Scale

The Warwick–Edinburgh Mental Wellbeing Scale (WEMWBS) is a self‐report measure consisting of 14 positively worded items that cover thoughts and feelings over the previous 2 weeks. Items are scored from 1 = *none of the time* to 5 = *all of the time*; therefore, scores can range from 14 to 70. Scores of over 60 are considered to be high. Its reliability and validity have been shown to be good (Stewart‐Brown et al., 2011); for the current sample, Cronbach's alpha was .93. For this analysis, individuals received a binary rating of high wellbeing (*yes/no*) as well as their total wellbeing score, with higher scoring denoting greater wellbeing (WEMWBS; Tennant et al., 2007).

### Procedure

Participants from the Depression Case Control study who had given permission to be re‐contacted were approached initially to enable analysis of the wider study of negative life events in relation to pre‐determined clinical history status (DeCC study; see Korszun et al., 2004 for full details). Student participants were recruited through the university by emails and letters outlining the study and providing log on details. Participants accessed the online life events measure and online versions of the questionnaire measures using a unique logon. All participants provided informed consent and the study was conducted with the ethical approval of IRAS and the University of Middlesex Psychology Ethics Committee.

### Analyses

The derived binary scales were used to establish the frequency of highly positive events, having two or more people close, attachment style and high wellbeing in the student, midlife clinical and midlife control groups. Chi‐square analyses using these variables determined whether any group differences were significant. One‐way analysis of variance (ANOVA) were calculated with attachment style as the factor and the continuous variables of wellbeing total score, total number of positive life events, total number of close relationships, and mean levels of confiding entered as the dependent variables to assess mean differences. The data were non‐normally distributed, however ANOVA and the *F*‐test are robust against Type I error, even at severe departures from normality (Blanca et al., 2017). Intercorrelations were calculated between the continuous variables total number of positive life events, wellbeing total score, total number of people close, attachment total score, and age using Pearson's correlations.

Lastly, to establish the relationships between positive events, secure attachment, and wellbeing, the variables were mean centered and a hierarchical multiple regression model was run with wellbeing as the dependent measure. To control for sex, age, and group status, these were added in the first step. The total number of positive events and secure attachment score were added in the second step and mean level of relational confiding and number of close others were included in the third step to ascertain whether social support confounds the association between positive life events, attachment, and wellbeing. An interaction term between attachment and positive life events was added in the final step. Residual analysis demonstrated the errors were normally distributed. In order to aid interpretation of the interaction, simple slopes analysis was conducted.

## RESULTS

Overall, 236 people (48%) had experienced at least one highly positive life event in the last 12 months (range: 0–9). The wellbeing scores ranged 16–70 with 9% (43) of scores considered high. There were 196 (40%) participants with secure attachment style; attachment scores ranged 31–89 (mean = 58.77, *SD* = 10.76), and 59% (288) of participants had at least two people who they were close to.

The groups were compared on frequencies of highly positive events, high wellbeing, attachment style, and having two or more people close using chi‐square (see Table 1). The number of individuals in each group who had experienced at least one highly positive event was not significantly different. Wellbeing was significantly higher in the midlife control group, with low rates in the clinical and student groups (*p* < .0001). Secure attachment style and close relationships were similarly highest in midlife control participants but lowest in students, although in the case of close relationships this difference was not statistically significant (*p* > .05).

**TABLE 1:** Key Binary Variable Frequencies by Group

Binary variables	Total % (*n*)	Midlife control group *N* = 128% (*n*)	Midlife clinical group *N* = 75% (*n*)	Student group *N* = 287% (*n*)	χ^2^, *df*, *p*
Highly positive event (yes/no)	48 (236)	52 (67)	45 (34)	47 (135)	1.28, 2, *p* = .53
Wellbeing (high vs. moderate/low)	43 (9)	21 (27)	4 (3)	5 (13)	32.86, 2, *p* < .0001
Secure vs. insecure attachment style	40 (196)	77 (98)	43 (32)	23 (66)	105.6, 2, *p* < .0001
2+ people close	59 (288)	64 (82)	59 (44)	56 (162)	2.12, 2, *p* = .35

An ANOVA was used to explore how secure attachment style was related to wellbeing scores, levels of confiding, and total number of positive events and close relationships (see Table 2). Individuals with a secure attachment style had significantly higher wellbeing (*F*[1, 486] = 122.81, *p* < .001), reported significantly more positive events (*F*[1, 486] = 4.47, *p* = .04), had more close relationships (*F*[1, 486] = 17.36, *p* < .001), and higher levels of confiding (*F*[1, 486] = 38.59, *p* < .001).

**TABLE 2:** Mean (SD) Values and one‐way ANOVA Showing the Effects of Attachment Style on Wellbeing Score, Mean Level of Confiding, Number of Positive Events, and Number of People Close

	Total mean (*SD*)	Secure attachment mean (*SD*)	Insecure attachment mean (*SD*)	*F*	*p*
Wellbeing	47.31 (9.83)	52.67 (8.4)	43.68 (9.1)	122.81	.001
Total positive events	0.91 (1.31)	1.07 (1.4)	0.81 (1.2)	4.47	.035
People close	1.83 (1.43)	2.16 (1.1)	1.62 (1.6)	38.59	.001
Confiding	1.14 (0.83)	1.4 (0.7)	0.96 (0.9)	17.36	.001

Correlations demonstrated that there was no significant association between age and number of positive events experienced (see Table 3). Wellbeing increased with age and was associated with having more positive life events and more close relationships. Secure attachment style score was associated with age, number of positive events, number of people close, and wellbeing (see Table 3).

**TABLE 3:** Correlations Between Variables

	Positive events	Wellbeing	People close	Secure attachment score
Positive events	–			
Wellbeing	.12**	–		
People close	.21**	.11**	–	
Secure attachment	.14**	.57**	.18**	–
Age	−.03	.23**	.03	.46**

** *p* < .01.

Multiple regression demonstrated that experiencing a positive event tended to increase the wellbeing score by 0.65 points on the scale, whilst on average, individuals with a secure attachment style scored approximately 7 points higher on wellbeing. The addition of controlling for level of confiding and close others did not explain variability above and beyond the contributions of attachment and positive events (see Table 4).

**TABLE 4:** Model of Positive Life Events and Insecure Attachment on Wellbeing

Step 1^a^	*B*	(*SE*)	β	*t*
Constant	4.86***	0.78		6.20
Age	0.42***	0.04	.82	9.62
Gender	0.96	1.03	.04	0.94
Group	–9.10***	1.10	–.70	−8.31
Step 2^b^				
Constant	0.98	0.87		1.13
Total positive events	0.65*	0.29	.09	2.25
Secure attachment	7.04***	0.88	.46	8.04
Age	0.28***	0.04	.55	6.42
sex	1.29	0.96	.06	1.34
Group	−7.39***	1.04	−.57	−7.10
Step 3^c^				
Constant	1.06	0.87		1.22
Total positive events	0.57*	0.30	.08	1.95
Secure attachment	6.87***	0.89	.35	7.70
Confiding	–0.72	0.66	–.06	–1.09
People close	0.70	0.38	.10	1.84
Age	0.29***	0.04	.57	6.60
sex	1.58	0.97	.07	1.63
Group	−7.54***	1.04	−.58	−7.23
Step 4^d^				
Constant	1.03	0.87		1.18
Total positive events	0.03	0.40	.00	0.08
Secure attachment	6.79***	0.89	.34	7.62
Total positive events * Secure attachment	1.15*	0.58	.11	1.97
Confiding	–0.66	0.66	–.06	–1.01
People close	0.73	0.38	.11	1.93
Age	0.29***	0.04	.57	6.60
Gender	1.44	0.97	.06	1.48
Group	−7.48***	1.04	−.57	−7.19

*** *p* < .001.

* *p* < .05.

^a^ *R* ^2^ = .171, *F*(3, 476) = 33.87, *p* < .001.

^b^ *R* ^2^ = .281, F(5, 474) = 38.38, *p* < .001.

^c^ *R* ^2^ = .283, F(7, 472) = 27.99, *p* < .001.

^d^ *R* ^2^ = .287, F(8, 471) = 25.12, *p* < .001.

**FIGURE 1:**
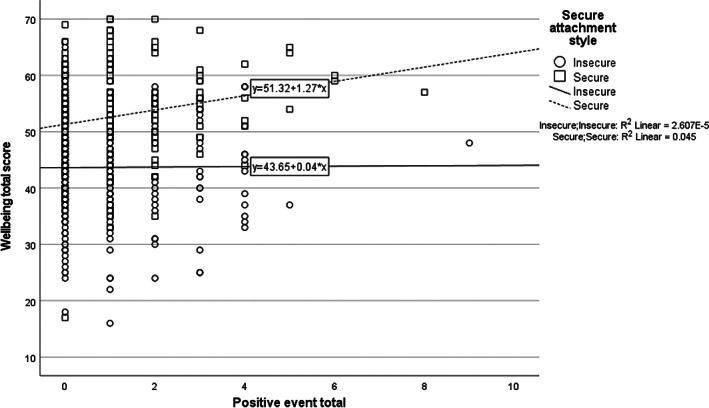
Predicted wellbeing score by attachment style and number of positive events with fit lines added by attachment style

The interaction between attachment style and number of positive events was significant, meaning the increase in wellbeing after experiencing a positive event was different depending on attachment style. Tests of simple slopes found a significant association between positive events and wellbeing for secure attachment style (*B* = 1.27, *p* = .003) but not for insecure attachment (*B* = .04, non‐significant). An individual with a secure attachment who had experienced an average number of positive events should expect to score approximately 43 on wellbeing, whilst an insecurely attached individual would expect to score approximately 37. For every positive event experienced, a securely attached individual's wellbeing score increased by 1.18 points, while an insecurely attached individual's score only increased by 0.03 (see Figure 1). Both the models explained approximately 28% of the variance in wellbeing.

## DISCUSSION

This study examined an accumulative positivity model investigating how secure attachment style interacts with positive life events in regards to increased wellbeing. Secure attachment style and positive life events were both significantly associated with increased wellbeing. There was a significant interaction between attachment style and positive events, with securely attached individuals experiencing a significantly greater increase in wellbeing after a positive life event. These results suggest securely attached individuals are better able to take advantage of positive life events than insecurely attached individuals and therefore experience more of an increase to their subjective wellbeing.

As psychosocial models of vulnerability suggest (Allen et al., 1998; DiTommaso et al., 2003), insecurely attached individuals had lower levels of social support; they reported significantly fewer close relationships and lower levels of confiding within these relationships. However, level of confiding and close others did not contribute significantly to the model or explain variability above and beyond the contributions of attachment style and positive events. This implies that although social support is important for wellbeing and lower disorder when experiencing negative events (Brown et al., 1990), it is not sufficiently effective in the experience of raised wellbeing after positive life events. This is potentially because those who have an insecure attachment style are not able to utilize the support that may actually be around them.

Recently, cognitive factors have been considered one of the major mechanisms linking early childhood experiences and depressive symptoms later in life (Fuhr et al., 2017). Indeed, there is evidence to suggest that how people perceive, interpret, and think about life events is linked to their general happiness (Lyubomirsky & Ross, 1999; Lyubomirsky & Tucker, 1998). In particular, certain attitudes which are associated with insecure attachment have been found to mediate the relationship between attachment and lack of wellbeing, this includes hopelessness (Lavy & Littman‐Ovadia, 2011), low self‐esteem (Fuhr et al., 2017), and fragility of happiness; the belief that happiness can cause bad things to happen and that when happiness is achieved it will not last long (Joshanloo, 2018). Thus, these beliefs may prevent insecurely attached individuals from feeling an increase in wellbeing after experiencing a positive life event. Indeed, there is some evidence suggesting securely attached individuals savor positive experiences more whilst insecure individuals minimize them (Gentzler et al., 2010; Gentzler et al., 2014). This may cause securely attached individuals to construe positive life events differently, maximizing their impact to create a differential association with wellbeing.

This interpretation is supported by studies tracking back memory processes to early mother–child interaction (McDonnell et al., 2016). In examining mothers reminiscing with their children, those with insecure attachment elaborated less on relating memories, focused on negative memories, and were less likely to impart those positive (McDonnell et al., 2016). This links with Belsky's (2001) summary of insecure individuals being less likely to both access and generalize positive memories, for example, those of prior coping, to aid positivity about future coping. Such inability to absorb positive information, whether from events or from social interactions, can seriously limit recovery and wellbeing processes in those vulnerable. Thus providing a greater likelihood of succumbing to clinical disorder, but a much lower likelihood of maintaining wellbeing at other times.

The clinical and student groups had lower wellbeing than the midlife group. In fact the students who were randomly selected had the same rate (5%) of high wellbeing as those midlife individuals (4%) with a recurrent history of depression. This corresponds with evidence suggesting levels of psychological distress amongst university students is high and greater than similar age groups within the general population (Unite, 2016). Students also demonstrated the lowest rates of secure attachment style, whilst wellbeing and secure attachment style scores both increased with age and were associated with having more positive events and more close relationships. Not many studies have addressed the stability of attachment in adulthood. However, some evidence supports a move to secure attachment as individuals age (Chopik et al., 2019), with variables such as fewer negative life events and relationship satisfaction being associated with this change towards attachment security (McConnell & Moss, 2011). However, the cross‐sectional nature of the current study means that we cannot determine the direction of any relationships found here.

These findings have some implications for clinical work; individuals with depression are often encouraged to undertake behavioral activation whereby they engage in activities and events that they are likely to enjoy. However, as individuals with an insecure attachment style derive less of an increase to their wellbeing from experiencing positive events, core beliefs and attitudes around positive experiences might need to be engaged with at the same time to maximize the benefits from these sorts of interventions. Thus, therapeutic work is needed not only to reduce negative interpretations but to increase positive interpretations of events and of social interactions. Future research would benefit from exploring the connections between positive life events, cognitive style, attachment, and subsequent wellbeing.

Other implications are for universities and their counselling services. Students had low levels of wellbeing and confiding relationships. Although vulnerability in UK student populations is now increasingly recognized with both academic (Macaskill & Denovan, 2013) and policy (National Collaborating Centre for Mental Health [Great Britain] et al., 2011) attention, this suggests more may need to be done both to increase mental health and decrease social isolation. Furthermore, the findings here suggest that whilst students did not differ in the number of positive events they experienced, the lower rates of secure attachment found amongst students and younger age groups more generally may make them less likely to be able to capitalize on these.

This study has some limitations; it is cross‐sectional and therefore cannot determine the directionality of the relationships. It is possible that those with higher wellbeing remember events more positively (Lyubomirsky & Ross, 1999) rather than positive events leading to increased wellbeing. The sample is reasonably small and selected; it warrants more research with a sample that is more representative of the general population. Measures used are self‐report, although the online life events tool is extensive and elaborated thus encouraging more objective reporting (Bifulco et al., 2019a). This study does not explore how long any changes to wellbeing last, which could be problematic as some have found the impact to be brief, suggesting positive life events may only change wellbeing temporarily (Suh et al., 1996). However, in the present study positive events from over a 12‐month period were utilized in relation to a measure of wellbeing at point of assessment suggesting the association may be reasonably sustained.

Despite these limitations, this study extends our understanding of psychosocial models of wellbeing by demonstrating how positive life events are associated with wellbeing and highlights the importance of secure attachment in moderating this process. This could be viewed as an accumulative positivity effect. Future research could build on this work by exploring if distinct types of cognitive style are more likely to lead to changes in wellbeing and for which particular attachment styles.

## DISCLOSURE OF CONFLICT OF INTEREST

None declared.

## ETHICS STATEMENT

All participants were provided with information about the study and consented to take part. Ethical approval was granted from the university's Psychology Department's Ethics Committee.
